# Targeting Non-Coding RNAs in Plants with the CRISPR-Cas Technology is a Challenge yet Worth Accepting

**DOI:** 10.3389/fpls.2015.01001

**Published:** 2015-11-19

**Authors:** Jolly Basak, Chandran Nithin

**Affiliations:** ^1^Department of Biotechnology, Visva-Bharati UniversitySantiniketan, India; ^2^Computational Structural Biology Lab, Department of Biotechnology, Indian Institute of Technology KharagpurKharagpur, India

**Keywords:** miRNA, lncRNA, CRISPR-Cas, NHEJ, homologous recombination, off-target mutation

## Abstract

Non-coding RNAs (ncRNAs) have emerged as versatile master regulator of biological functions in recent years. MicroRNAs (miRNAs) are small endogenous ncRNAs of 18–24 nucleotides in length that originates from long self-complementary precursors. Besides their direct involvement in developmental processes, plant miRNAs play key roles in gene regulatory networks and varied biological processes. Alternatively, long ncRNAs (lncRNAs) are a large and diverse class of transcribed ncRNAs whose length exceed that of 200 nucleotides. Plant lncRNAs are transcribed by different RNA polymerases, showing diverse structural features. Plant lncRNAs also are important regulators of gene expression in diverse biological processes. There has been a breakthrough in the technology of genome editing, the CRISPR-Cas9 (clustered regulatory interspaced short palindromic repeats/CRISPR-associated protein 9) technology, in the last decade. CRISPR loci are transcribed into ncRNA and eventually form a functional complex with Cas9 and further guide the complex to cleave complementary invading DNA. The CRISPR-Cas technology has been successfully applied in model plants such as *Arabidopsis* and tobacco and important crops like wheat, maize, and rice. However, all these studies are focused on protein coding genes. Information about targeting non-coding genes is scarce. Hitherto, the CRISPR-Cas technology has been exclusively used in vertebrate systems to engineer miRNA/lncRNAs, but it is still relatively unexplored in plants. While briefing miRNAs, lncRNAs and applications of the CRISPR-Cas technology in human and animals, this review essentially elaborates several strategies to overcome the challenges of applying the CRISPR-Cas technology in editing ncRNAs in plants and the future perspective of this field.

## Micro RNAs and Long Non-Coding RNAs

**FIGURE 1:**
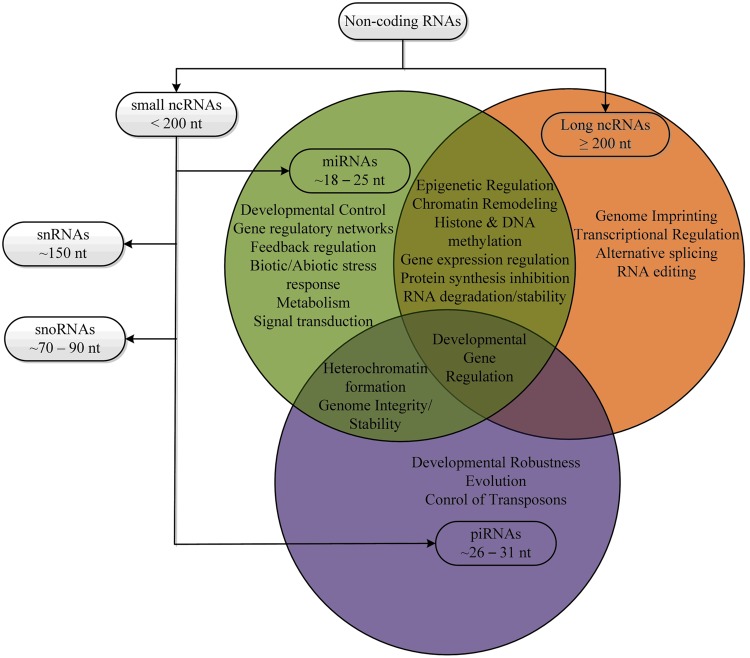
**Classification and major functions of various non-coding RNAs**.

MicroRNAs (miRNAs) are small endogenous non-coding RNAs (ncRNAs; Ambros, 2001) of 20 to 24-nucleotide in length, originating from long self-complementary precursors (Bartel, 2004; Nithin et al., 2015). Mature miRNAs regulate gene expression in two ways; (i) by inhibiting translation or (ii) by degrading coding mRNAs by perfect or near-perfect complement with the target mRNAs (Carrington and Ambros, 2003; Djuranovic et al., 2011; Nithin et al., 2015). The majority of plant target mRNAs contain a single miRNA-complementary site where corresponding miRNAs perfectly complement, thereby cleaving the target mRNAs (Kidner and Martienssen, 2005; Nithin et al., 2015). During the last decade, several studies have confirmed that plant miRNAs are directly involved in the developmental processes such as root development, seed germination, morphogenesis, vegetative and reproductive phase change, and flowering initiation (Jones-Rhoades et al., 2006; Jung et al., 2009; Nodine and Bartel, 2010; Wu et al., 2011; Yang et al., 2011; Nithin et al., 2015). Moreover, plant miRNAs are the key players of gene regulatory networks, regulating diverse biological processes like metabolism, biotic and abiotic stress response, signal transduction, protein degradation, siRNA pathway feedback regulation, and maintenance of genome integrity (Mallory and Vaucheret, 2006; Bushati and Cohen, 2007), (**Figure 1**).

In contrast, long ncRNAs (lncRNAs) are a large and diverse class of transcribed ncRNAs whose length exceed that of 200 nucleotides, localized within the nucleus with few exceptions in the cytosol (Louro et al., 2008; Mercer et al., 2008). Compared to coding mRNAs, lncRNAs have shorter length, lower abundance, are restricted to particular tissues or cells and less frequently conserved between species (Derrien et al., 2012). Generally, lncRNA biogenesis is very similar to coding mRNAs (Dieci et al., 2007). LncRNAs can originate from intronic, exonic, intergenic, intragenic, promoter regions, 3′- and 5′ UTRs, enhancer sequences and can transcribe bidirectionally (Nie et al., 2012). LncRNAs also possess post-transcriptional modifications (Consortium et al., 2005). LncRNAs play a key role in regulating important biological processes (**Figure 1**) by one of the following ways; enhancing the accessibility of target site to RNA polymerases, binding to the promoter DNA of the target gene forming a RNA-dsDNA triplex, inhibiting RNA polymerase activities and regulating transcription factors (Lipshitz et al., 1987; Nguyen et al., 2001; Willingham et al., 2005; Martianov et al., 2007; Hirota et al., 2008; Mariner et al., 2008). Moreover, lncRNAs play a role in post-transcriptional modulations of mRNA processing. Distinct classes of lncRNAs in multiple species are increasingly being recognized, emerging as important regulators of gene expression in various biological processes (Zhu and Wang, 2012; Fatica and Bozzoni, 2014). Plant lncRNAs are transcribed by different RNA polymerases showing diverse structural features (Mercer and Mattick, 2013).

## The CRISPR-Cas Technology

**FIGURE 2:**
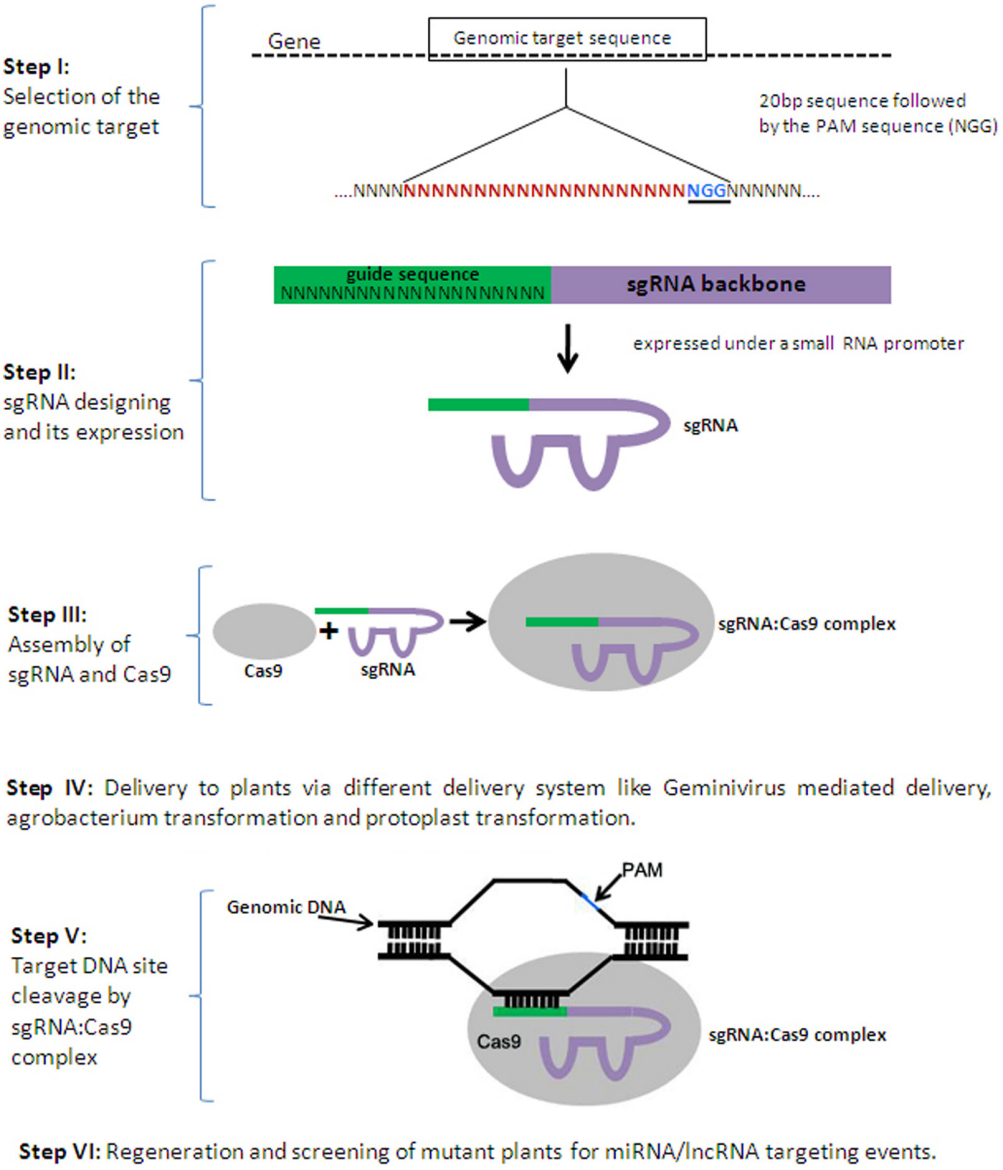
**Series of events to generate a clustered regulatory interspaced short palindromic repeats/CRISPR-associated (CRISPR-Cas) mutagenised plant.** CRISPR-Cas mediated genome engineering in plants requires ‘single guide’ RNA (sgRNA) and CRISPR-associated protein 9 (Cas9). Specially designed sgRNA are expressed under a small RNA promoter and transfected along with a Cas9 expression plasmid, to form a complex which targets complementary DNA adjacent to the protospacer adjacent motif (PAM). sgRNA:Cas9 complex generates a double strand break (DSB) that may either be repaired precisely (without any effect) or imperfectly leading to a mutation (indel) in the genomic target sequence.

In the last decade, development of sequence-specific nucleases like zinc finger nucleases (ZFNs) and transcription activator-like effector nucleases (TALENs) have revolutionized the process of conventional plant breeding by successfully generating efficient genetic variants of crop plants (Kim et al., 1996; Christian et al., 2010; Miller et al., 2011). These nucleases modify the genome by generating double strand breaks (DSBs), which are then repaired through non-homologous end joining (NHEJ) or homologous recombination (HR; Bibikova et al., 2003; Carroll, 2011; Reyon et al., 2012; Belhaj et al., 2015). ZFNs and TALENs are composed of programmable, sequence-specific DNA-binding modules fused with FokI nuclease domain (Urnov et al., 2010; Joung and Sander, 2013). However, it is quite painstaking and expensive to design and construct large modular proteins and it is also associated with a high rate of failure. Recently, another breakthrough technology for genome editing, the clustered regulatory interspaced short palindromic repeats/CRISPR-associated (CRISPR-Cas) technology, has been developed (Barrangou et al., 2007; Horvath and Barrangou, 2010; Cong et al., 2013). CRISPR loci are variable short spacers separated by short repeats, which are transcribed into ncRNAs and eventually form a functional complex with CRISPR-associated protein 9 (Cas9) and further guide the complex to cleave complementary invading DNA (**Figure 2**), (Mali et al., 2013; Hsu et al., 2014; Belhaj et al., 2015). The guide RNA/Cas9 nuclease complex overcomes some of the limitations of previous tools. 20 base pair guide RNAs (gRNA) are easy to design and can target the Cas9 protein to almost any desired region in the genome to bind to its DNA target by Watson-Crick base-pairing (Gilbert et al., 2013; Bortesi and Fischer, 2015). Target recognition is dependent on the so-called ‘protospacer adjacent motif’ (PAM), for which the consensus sequence, NGG, is adjacent to the 3′ end of the 20 bp target (Anders et al., 2014; Sander and Joung, 2014; Bortesi and Fischer, 2015). After the initial development of a programmable CRISPR-Cas technology, it has been rapidly applied to achieve efficient genome editing in human cell lines, zebrafish, mouse, rice, and *Arabidopsis* (Hwang et al., 2013a,b; Doench et al., 2014; Jiang et al., 2014b; Zhou et al., 2014; Ho et al., 2015). The small size of the guide RNA (20 bp) allows the co-delivery of multiple ‘single guide’ RNAs (sgRNA) with Cas9 to the cell, making it feasible to simultaneously edit more than one target sequence at the same time. The ease and robustness of this technology makes it an attractive genome editing tool for plant biology.

The CRISPR-Cas technology has been successfully applied in model plants *Nicotiana benthamiana, N. tabacum*, and *Arabidopsis*, and crops, such as wheat, maize, rice, sorghum, tomato, and sweet orange (Jiang et al., 2013; Brooks et al., 2014; Feng et al., 2014; Jia and Wang, 2014; Shan et al., 2014; Gao et al., 2015). In both *Arabidopsis* and rice, the percentage of regenerated plants containing a CRISPR/Cas9 transgene with detectable mutation, has been reported as high as 90% (Miao et al., 2013; Feng et al., 2014). Several studies demonstrated the Mendelian heritability of CRISPR-Cas-induced mutations in *Arabidopsis*, rice, and tomato (Brooks et al., 2014; Feng et al., 2014; Jiang et al., 2014b). However, all these studies are focused on protein coding genes. Information about targeting non-coding RNAs is scarce. Hitherto, the CRISPR-Cas technology has been exclusively applied in human cell lines, mouse, or zebrafish to knockout miRNA genes or lncRNA genes (Xiao et al., 2013; Han et al., 2014; Zhao et al., 2014). However, there is no report of engineering miRNA or lncRNA genes in plants using the CRISPR-Cas technology.

## Application of the CRISPR-Cas Technology in Targeting Non-Coding Genes in Animals and Human Cell Lines

Jiang et al. (2014a) applied the CRISPR-Cas technology to investigate the function of a specific human miRNA, miR-93. Generally, the 5′ end of the miRNA is precisely cleaved by Drosha and contains the seed region, which is critical for target recognition. Accordingly, Jiang et al. (2014a) targeted the 5′ region of human miR-93 genes in HeLa cells. Several small indels were induced in the targeted region containing the Drosha processing site and seed sequences. Surprisingly, it was found that even a single nucleotide deletion led to the complete knockout of the target miRNA with high specificity (Jiang et al., 2014a). Phenotype analysis confirmed the functional knockout while structural analysis revealed the impaired biogenesis process (Jiang et al., 2014a). Furthermore, qRT-PCR confirmed the absence of mature miR-93 in mutated cells (Jiang et al., 2014a). Using the CRISPR-Cas as a novel tool, Jiang et al. (2014a) showed the depletion of a single miRNA by introducing indels at the 5′ end of its mature sequence and confirmed that the alteration of a single or a few nucleotides in the specific genomic sequence not only depletes miRNA, but also retards Drosha processing.

Ho et al. (2015) carried out the knockdown of non-coding genes using the CRISPR-Cas technology in human cell lines. Two miRNAs, miR-21 and miR-29a, and three lncRNAs UCA1, lncRNA-21A, and AK023948 were selected. Either a single gRNA or three gRNAs together were used to target miR-21 (Ho et al., 2015). It was found that each of the three individual gRNAs or their combination, produced mismatched bands with targeting frequency of 17–39%, suggesting that this approach is robust and miR-21 sequence is altered (Ho et al., 2015). One of the challenges for knocking out of non-coding genes is that a small deletion or insertion generated by the standard CRISPR-Cas technology may not necessarily lead to functional loss of a given non-coding gene due to absence of an open reading frame, especially in polyploidy human cell lines (Ho et al., 2015). To overcome this challenge, Ho et al. (2015) adopted a selection system that allows the integration of marker genes into the genome through HR, and showed that HR-mediated targeting efficiency can be further improved by suppression of the NHEJ pathway.

Zhao et al. (2014) reported a convenient and efficient miRNA inhibition strategy employing the CRISPR-Cas technology to knock out the non-coding genes in murine cells. Two miRNAs, miR-21 and miR-30a, were targeted. Specifically designed gRNAs were used to cut the miRNA genes at a single site by Cas9, resulting in knockdown of the miRNAs in murine cells (Zhao et al., 2014). Zhao et al. (2014) established that inactive Cas9 can reversibly prevent the expression of both monocistronic miRNAs and polycistronic miRNA clusters when a modified CRISPR interference system (CRISPRi) is used. CRISPR-CRISPRi is also capable of suppressing the genes in porcine cells.

Pefanis et al. (2015) applied the CRISPR-Cas technology to ablate the RNA-degradation machinery in B-cells and embryonic stem cells by conditional mutagenesis of the RNA-exosome, resulting in the identification of numerous lncRNAs and enhancer-RNAs (eRNAs) with promising functionality. Surprisingly, it was found that the RNA-exosome regulates the levels of divergently transcribed eRNAs by promoting co-transcriptional silencing, thereby preventing the persistence of detrimental chromatin structures that can lead to genomic instability (Pefanis et al., 2015). Moreover, Pefanis et al. (2015) discovered a distal divergent eRNA-expressing element (lncRNA-CSR) which is engaged in long-range DNA interaction and regulate super-enhancer function. It was found that CRISPR-Cas9-mediated ablation of this lncRNA-CSR transcription decreases its chromosomal looping-mediated association with super-enhancer (Pefanis et al., 2015). Thus, the CRISPR-Cas technology was successfully applied to understand the mode of long-range chromatin regulation.

## Challenge of the CRISPR-Cas Technology in Targeting Non-Coding Genes in Plants

### Hurdle I: Quest for an Effective Delivery System in Plants

Precise modification of plant genomes, a basic requirement for gene function studies and crop improvement programs, can be achieved by introducing targeted DSBs, which thereby activate two main repair pathways; NHEJ and HR. NHEJ repair mechanism is imprecise. It generates indels at the cut site, resulting in endogenous gene disruption or mutagenesis (Lloyd et al., 2005; Zhang et al., 2010; Baltes et al., 2014; Belhaj et al., 2015). Alternatively, HR uses sister-chromatid or homologous-chromosome for template-directed repair. With an exogenous supply of repair template, gene replacement, or targeted gene insertion in HR is likely to be perfect (Bibikova et al., 2003; Baltes et al., 2014; Belhaj et al., 2015). However, targeted modification of plant genomes is still a challenge due to ineffective methods of delivery systems to plant cells. Although Protoplast-transformation yields higher gene targeting frequency compared to physical-method of genetic transformation or *Agrobacterium-*mediated transformation, yet the plant regeneration frequency is very low (Shukla et al., 2009; Zhang et al., 2010).

To overcome this, Baltes et al. (2014) developed an efficient and facile vector system using Geminiviruses. Geminiviruses are a large family of plant viruses with circular, single-stranded DNA genomes that replicate through double-stranded intermediates. Baltes et al. (2014) used the model plant *Arabidopsis* and established that a Geminiviral-sequence can function as a template for homologous repairing of a DSB. Deconstructed bean yellow dwarf virus was used to deliver CRISPR-Cas through *Agrobacterium-*mediated transformation and cells fixed the generated DSBs through NHEJ (Baltes et al., 2014). Moreover, precise gene targeting was possible through the delivery of CRISPR-Cas and deconstructed Geminiviruses as repair templates and the generated DSBs were fixed by cells through homology-dependent repair (Baltes et al., 2014). Baltes et al. (2014) established that gene targeting and repairing frequency is higher for Geminivirus-based delivery compared to other methods and targeted cells rapidly regenerate into plantlets with precise genomic modifications. Success of the CRISPR-Cas technology largely depends on the effective delivery of the components in plant. Thus, application of Geminivirus-mediated delivery in this technology can improve its success rate. Moreover, as Geminiviruses infect both monocots and dicots, the CRISPR-Cas technology employing Geminivirus-mediated delivery can engineer a vast range of crops.

### Hurdle II: Off-target Mutations in Plants

In spite of being a powerful genome editing tool, the CRISPR-Cas technology has several drawbacks, of which the most alarming is the off-target mutation. Several strategies were developed to reduce off-target genome editing; careful designing of the gRNA being the most promising one. Target recognition in the CRISPR-Cas technology takes place by Watson–Crick base pairing, allowing off-target sites to be predicted more accurately by sequence data analysis (Cho et al., 2014; Bortesi and Fischer, 2015). Moreover, due to easy reprogramming, gRNAs can be tested for off-target effects rapidly and inexpensively (Bortesi and Fischer, 2015). Hsu et al. (2013) parallely studied more than 700 sgRNAs to understand the targeting specificity. Based on the study, the authors developed a number of guidelines and online tools to facilitate the selection of unique target sites as well as off-target analyses in well-characterized organisms including several plants. The length of the gRNA also plays a major role in off-target mutation.

Cho et al. (2014) established that gRNAs with two additional guanidine residues at the 5′ end can avoid off-target sites more efficiently than normal gRNAs; however, elongated gRNAs are slightly less active at on-target sites. In contrast, Fu et al. (2014) showed that truncated gRNAs having shorter regions of target complementarity (17 nucleotides in length) can reduce undesired mutagenesis at some off-target sites by 5,000-fold or more, without affecting on-target genome editing efficiencies. Here, truncations make the RNA–DNA complex more sensitive. Specificity can also be controlled by optimizing nuclease expression, as high concentrations of gRNA and Cas9 can advance off-target mutations (Fujii et al., 2013; Hsu et al., 2013; Pattanayak et al., 2013). To improve DNA cleavage specificity, Guilinger et al. (2014) generated fusions of catalytically inactive Cas9 and FokI nuclease (fCas9). The authors showed that proper cleavage of DNA by fCas9 requires union of two fCas9 monomers that concurrently bind target sites which are ∼15 or 25 base pairs apart. A comparison between fCas9 and wild-type Cas9 with efficiency similar to that of paired Cas9 ‘nickases’ (engineered variants that cleave only one DNA strand per monomer) showed that fCas9 can modify target DNA sites with >140-fold higher specificity (Guilinger et al., 2014; Tsai et al., 2014). The specificity of fCas9 was at least fourfold higher than that of paired nickases at loci with highly similar off-target sites (Guilinger et al., 2014). Fu et al. (2014) proved that combining the truncated gRNAs and Cas9 nickase approaches together could potentially increase the specificity even further.

Thus to overcome off-target mutations, the following strategies should be considered; proper selection of target sequence with high specificity, careful truncation or elongation of gRNAs and construction of intelligent mutations in Cas9. Moreover, on-target mutations often precede off-target mutations, resulting in loss of novel mutations after regeneration. Thus, a short selection period for calli during redifferentiation can prevent off-target mutations.

### Hurdle III: Targeting miRNAs and lncRNAs in Plants

Knockdown or knockout of ncRNA genes compared to protein coding genes using the CRISPR/Cas technology is a challenge. Small non-coding gene knockdown/knockout is complicated for CRISPR-Cas9 because of the limited design space for targeting the non-coding genes without disturbing genes in the vicinity (Barrangou et al., 2015). This is particularly a problem for silencing miRNAs as many of them are encoded within introns of protein-coding host genes. However, CRISPR-Cas9-mediated knockout of miRNAs have the potential to be more efficient by targeting miRNA genes at multiple sites like promoter and hairpin (Barrangou et al., 2015). Another way of promoter targeting can be done by using a catalytically inactive Cas9 in combination with sgRNA (CRISPRi) for precise interference of the transcriptional machinery (Qi et al., 2013). Moreover, CRISPR-Cas can be designed to target both the 5′ or 3′ arm of the mature miRNA. Another avenue of the CRISPR-Cas technology is its successful application in generating mutant miRNA binding sites in target genes, thereby verifying miRNA targeting (Bassett et al., 2014).

Even though lncRNAs have molecular weight comparable to that of protein coding genes, lack of ORFs for translation makes the commonly used approaches of the CRISPR-Cas technology limited for targeting lncRNAs. Han et al. (2014) developed an efficient one-step strategy to explore the potentiality of the CRISPR/Cas9 technology to generate large genomic deletions of lncRNAs in mice by targeting the maternally expressed lncRNA, Rian, on chromosome 12 (Han et al., 2014). Paired sgRNAs can be accurately used to generate large deletions amounting to 23 kb and combination of multiple sgRNAs can increase this deletion efficiency up to 33% (Han et al., 2014). In a similar manner, the CRISPR-Cas technology can be designed for plant lncRNAs by using paired sgRNAs or combining multiple sgRNAs.

## Conclusion and Future Perspective

Based on the published reports on plant applications, the CRISPR-Cas technology with its enormous potential as a straightforward genome editing tool has been anticipated as a routine technique for targeted gene knockdown/knockout in plants. However, its application in editing non-coding RNAs in plants is still nascent. Several strategies have been discussed to overcome the challenges of applying the CRISPR-Cas technology in editing ncRNAs in plant systems. The analysis of the outcome of application of these strategies in plants, through real experiments, will help in designing new improved strategies that will further improvise the CRISPR-Cas technology to engineer ncRNAs.

During the last decade, ncRNAs of all kinds have gained global attention as potentially novel and vital regulators of biological mechanisms, including developmental processes and diseases, but knowledge of the modus operandi is still surprisingly limited. Successful application of the CRISPR-Cas technology in editing ncRNAs in plant systems will help interpret and decipher their mode of action, thus opening a new avenue in science.

## Conflict of Interest Statement

The authors declare that the research was conducted in the absence of any commercial or financial relationships that could be construed as a potential conflict of interest.
